# Psychiatric and Cognitive Aspects of Phenylketonuria: The Limitations of Diet and Promise of New Treatments

**DOI:** 10.3389/fpsyt.2019.00561

**Published:** 2019-09-10

**Authors:** Killian Ashe, Wendy Kelso, Sarah Farrand, Julie Panetta, Tim Fazio, Gerard De Jong, Mark Walterfang

**Affiliations:** ^1^Neuropsychiatry Unit, Royal Melbourne Hospital, Melbourne, VIC, Australia; ^2^Statewide Adult Metabolic Service, Royal Melbourne Hospital, Melbourne, VIC, Australia; ^3^Melbourne Medical School, University of Melbourne, Melbourne, VIC, Australia; ^4^Melbourne Neuropsychiatry Centre, University of Melbourne and North-Western Mental Health, Melbourne, VIC, Australia; ^5^Florey Institute of Neuroscience and Mental Health, University of Melbourne, Melbourne, VIC, Australia

**Keywords:** phenylketonuria, psychiatric, anxiety, depression, cognitive function

## Abstract

Phenylketonuria (PKU) is a recessive disorder of phenylalanine metabolism due to mutations in the gene for phenylalanine hydroxylase (PAH). Reduced PAH activity results in significant hyperphenylalaninemia, which leads to alterations in cerebral myelin and protein synthesis, as well as reduced levels of serotonin, dopamine, and noradrenaline in the brain. When untreated, brain development is grossly disrupted and significant intellectual impairment and behavioral disturbance occur. The advent of neonatal heel prick screening has allowed for diagnosis at birth, and the institution of a phenylalanine restricted diet. Dietary treatment, particularly when maintained across neurodevelopment and well into adulthood, has resulted in markedly improved outcomes at a cognitive and psychiatric level for individuals with PKU. However, few individuals can maintain full dietary control lifelong, and even with good control, an elevated risk remains of—in particular—mood, anxiety, and attentional disorders across the lifespan. Increasingly, dietary recommendations focus on maintaining continuous dietary treatment lifelong to optimize psychiatric and cognitive outcomes, although the effect of long-term protein restricted diets on brain function remains unknown. While psychiatric illness is very common in adult PKU populations, very little data exist to guide clinicians on optimal treatment. The advent of new treatments that do not require restrictive dietary management, such as the enzyme therapy Pegvaliase, holds the promise of allowing patients a relatively normal diet alongside optimized mental health and cognitive functioning.

## Introduction

Phenylketonuria (PKU; OMIM 261600 and 261630) is a rare autosomal recessive and inborn error of metabolism (1). Caused by one of almost 1,000 gene variants (2), it is characterized by a variable deficiency in the activity of the phenylalanine hydroxylase (PAH) enzyme necessary for the conversion of the amino acid phenylalanine (Phe) to tyrosine (Tyr). The resultant accumulation of Phe throughout the body due to reduced PAH activity leads to significant neurodevelopmental sequelae including intellectual disability, growth retardation, and seizures (1), and is the most common biochemical cause of intellectual impairment (3). In addition, there are a range of neuropsychiatric and cognitive changes that can be associated with PKU at varying levels of hyperphenylalaninemia.

Phenylalanine is an essential amino acid, present in most natural proteinaceous foods, and is metabolized in the liver by the PAH system. The hydroxylation of Phe to Tyr requires tetrahydrobiopterin (BH4), iron, and molecular oxygen as co-factors (1). Defects in either PAH or the production or recycling of BH4 may result in hyperphenylalaninemia. The disorder was first discovered in 1934 by Følling who identified elevated Phe metabolites (phenylketones) in the urine of two intellectually disabled siblings, thus giving the disorder its name (4); the defect in PAH was isolated in 1953 (5), and a Phe-restricted diet was also shown to improve intellectual outcomes (6). In 1962, the Guthrie method to detect elevated Phe levels in dried blood spots was developed, which facilitated newborn screening of the disorder, facilitating early diagnosis and dietary management of PKU (7), and thus the prevention of many hundreds of thousands of cases of intellectual disability. The “Guthrie Test,” conducted by neonatal heel prick, was implemented widely across North America and Europe in the 1960s (7). It identifies affected infants at birth and has a 99.2% sensitivity and 99.9% specificity for those with classical and mild PKU (8), and has been demonstrated to have a favorable benefit–cost ratio (9).

Two Australian studies reported the incidence of PKU to be 1 in 11,226 (10) and 1 in 8,900, mirroring the figures found in other Caucasian populations (3). The reported incidence of PKU ranges from 1 per 13,500 to 1 per 19,000 newborns in the United States, and does vary by ethnic group, with incidence being higher in Caucasians and Native Americans and lower in Hispanics, Blacks, and Asians (11).

Depending on the genotype and severity of the enzyme defect, various forms of PKU with different clinical outcomes have been described (12). These can be classified on the basis of blood Phe levels at diagnosis and dietary Phe tolerance. The natural history of untreated PKU consists of progressive irreversible neurological impairment during infancy and childhood. The most common outcome is severe intellectual disability often associated with a “mousy” odor, eczema, and reduced hair, skin, and iris pigmentation. There is often growth retardation, microcephaly, and neurological signs including tremor and epilepsy. All untreated patients have behavioral problems including hyperactivity, stereotypy, and anxiety. The severity of the clinical phenotype directly correlates with blood phenylalanine levels that reflect the degree of enzymatic deficiency (1).

Until recently, a strict low-Phe diet, the use of medical foods consisting of Phe-free protein substitutes, and supplementation containing large neutral amino acids (LNAAs) such as Tyr were the only therapies available (13). Foods high in Phe, such as eggs, meat, poultry, fish, bread, and pasta, are largely eliminated. Dietary treatment has been shown to be very effective in the prevention of impaired cognitive development, but still has its shortcomings. Specific deficiencies of calcium, zinc, selenium, iron, and vitamin B12 were reported with the early formulas, as was growth delay (14).This diet was initially discontinued after 6 years of age, when the majority of significant neurodevelopment was thought to be complete (“early-treated” patients); this age was later increased to 12 years of age; however, these discontinuation points often varied between jurisdictions. Later epochs of treatment recommendations suggested cessation at early adulthood, with only a return to diet being necessitated during pregnancy. However, over time, it has been increasingly recognized that ongoing adherence to diet is associated with better cognitive and psychosocial outcomes throughout adulthood (“continuously treated” patients), as increasingly hyperphenylalaninemia has been recognized to not just impact upon neurodevelopment, but dynamic functioning within the adult brain (15). Current guidelines indicate that individuals with PKU should aim to maintain lifelong Phe levels between 120 and 360 µmol/L in the US, or up to 600 µmol/L in Europe, even though this is still up to a fivefold increase over Phe levels in non-PKU individuals (16, 17).

When treatment is instituted from birth, PKU patients are likely to have a normal intellectual quotient (IQ), but it is now apparent that there is often an IQ gap when compared to their non-PKU siblings, and these patients may have deficits in certain neuropsychological functioning, particularly executive function (18). They may also demonstrate elevated rates of neuropsychiatric illness, increasingly being recognized, which contribute to significant impairments in psychosocial functioning and quality of life in adults with treated PKU. This review aims to discuss the range of neuropsychiatric and cognitive manifestations associated with PKU and their management.

### Pathophysiology of PKU

Deficient PAH activity results in two key pathological states: hyperphenylalaninemia and hypotyrosinemia (19). High Phe competes with tyrosine, tryptophan, and other LNAAs at the L-type amino acid carrier (LAT1) at the blood–brain barrier (BBB), resulting in decreased concentrations of dopamine and serotonin in the brain (20). The transport of Phe across the BBB has been found to be highly variable, translating to heterogeneous clinical outcomes across individuals even when “on-diet” or with similar plasma Phe levels (11, 20).

**Figure 1:**
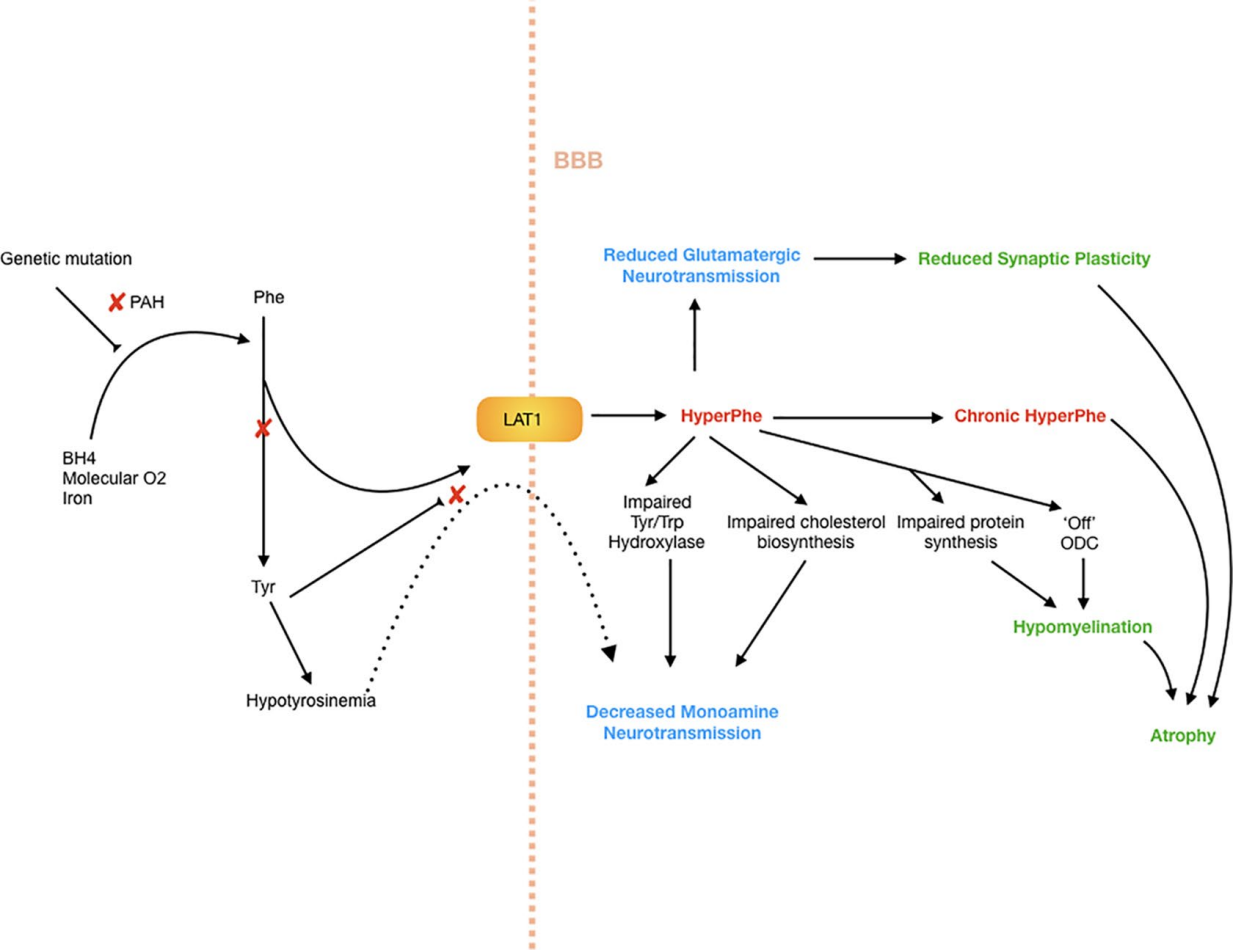
Neuropathophysiology of PKU. Decreased PAH activity results in hyperphenylalaninemia as well as hypotyrosinemia. The high Phe levels further restrict transport of tyrosine across the blood–brain barrier, as well as impairing tyrosine and tryptophan hydroxylase systems, as well as cholesterol synthesis, creating a hypomonoaminergic state in the brain, predominantly in frontal and limbic circuits where these neurotransmitters predominate. There is also reduced glutamatergic neurotransmission, which results in reduced synaptic plasticity, and in turn atrophy. Myelination is critically affected by high Phe levels, which impair protein synthesis, as well as switch oligodendrocytes to an “off” or non-myelinating state.

There are multiple possible downstream effects of hyperphenylalaninemia, for which the evidence and hypotheses are drawn from human and animal studies. These are summarized in broad categories below, with respect to neuropsychiatric and cognitive profiles. The mechanisms by which these effects occur are summarized in **Figure 1**.

### Neurotransmission and Protein Synthesis

Hyperphenylalaninemia causes dysfunction in neurotransmission and cerebral protein synthesis, *via* relative hypotyrosinemia, impaired transport of large amino acids into the brain, and impairment of other enzymatic systems. Not only is the biosynthesis of tyrosine reduced due to PAH deficiency, but also in a state of hyperphenylalaninemia, there is competitive binding at LAT1 carrier, resulting in Phe preferentially crossing the blood–brain barrier and a relative lack of other LNAAs including tryptophan and tyrosine, the building blocks of serotonin and dopamine (21). The low bioavailability of these LNAAs then has downstream effects on cerebral protein synthesis in evidence drawn from the PKU mouse model (21). Evidence from mice and human models also suggests that hyperPhe impairs the function of the tyrosine and tryptophan hydroxylase systems (22–24), as well as 3-hydroxy-3-methylglutaryl-coenzyme A reductase and thus impairing cholesterol biosynthesis (21), all of which effects a reduction in neurotransmitter production.

Glutamate is the most abundant neurotransmitter and plays an important role in synaptic plasticity, learning, memory, and modulation of function within the limbic system (25). In the PKU mouse model, hyperPhe was found to reduce glutamate transmission resulting in compensatory changes in the expression of the glutamate receptor (26). This occurred *via* competitive binding at the NMDA receptor, reduction in release of neurotransmitters, and competition for binding to sites on other receptors (such as AMPA and kainate glutamatergic receptors) (27). Importantly these findings were reversible, suggesting a degree of reversibility of neuropsychiatric symptoms seen in PKU. Glutamate has also been increasingly recognized for its role in stress-related conditions, including anxiety and depression. While acute stress can transiently increase glutamate output and transmission, chronic stress is associated with similar findings to those seen in the hyperPhe state, with reduced synaptic plasticity and attenuated glutamatergic neurotransmission (28).

### Synaptic and Dendritic Changes

Animal studies have greatly informed the field about the microstructural effects of hyperPhe. Treating newborn rodents with high levels of Phe during early life results in malformed dendritic trees (29, 30) and reduced neocortical synaptic density (31), possibly through the altered expression of cytoskeletal proteins (32). Studies of hippocampal cultures in the PAH^enu2^ mouse model treated with phenylalanine show a reduction in density of synapses, and length of dendrites (33), with synaptic morphological alterations (34). At least some of these morphological changes in the mouse model appear to be remediable by a specific nutrient combination, suggesting that attenuating synaptic deficits may be one potential mechanism of dietary intervention in PKU (30, 35).

### Dysmyelination

A number of studies have demonstrated abnormal myelination in PKU patients (36, 37). Bauman and Kemper undertook histoanatomic assessment of three brains in untreated, intellectually disabled adult PKU patients and found that there was pallor of myelin staining, particularly in areas that undergo significant postnatal myelination, as compared with those areas that undergo myelination shortly before or after birth, which were comparable to age matched controls (38). Later-myelinating areas include the axonal connections to the prefrontal cortex and other frontal and parietal regions, as well as cortico-hippocampal relay circuits, which undergo myelination in adolescence (39, 40). Elevations in Phe resultsin the non-competitive inhibition of HMG-CoA reductase, which impairs the production of cholesterol, one of the major lipids in the myelin membrane (41), and the reduction of LNAAs available for myelin-related proteins may compromise myelin synthesis further (42, 43).

These late-myelinating regions may be more vulnerable to suboptimal environmental conditions. Areas that undergo myelination later in development have smaller axons and fewer lamellae, and originate from oligodendrocytes that myelinate many more axons than those that undergo early myelination (44). In a review of the relationship of late-myelinating regions and neurodegeneration, Bartzokis postulated that this resulted in three broad sequelae—that these regions would have greater surface area exposed to the extracellular environment and thus greater vulnerability to a toxic (i.e., HyperPhe) environment, higher myelin density, and a greater impact when there is breakdown of these areas due to the thinner myelin sheaths (44). This model, while applied by Bartzokis to primary neurodegenerative disorders, has relevance to all neuropsychiatric disorders, and in combination with the specific areas involved in late myelination, gives reasonable account as to the high rates of psychiatric and cognitive symptoms seen in PKU.

Supporting this are data from both animal and human studies. In normal rats, chronic hyperPhe resulted in impaired myelination and axonal maturation (45). In the PAH^enu2^ mouse model, when exposed to higher levels of Phe, myelin-rich areas had the lowest rates of protein synthesis (46), and to further this, Dyer and colleagues found that when oligodendrocytes were exposed to high Phe levels, they adopt a “non-myelinating” phenotype, effectively switching off myelination (47). There is a strong relationship between dopamine signaling and myelination, in addition to axonal maturation. Myelination itself facilitates axonal maturation through triggering heavy phosphorylation of neurofilaments, cytoskeleton rearrangement, and axonal swelling beneath the compact myelin lamellae (48). These myelin/axonal interactions may up-regulate the production of enzymes involved in dopamine biosynthesis pathway such as tyrosine hydroxylase (TH), or the phosphorylation of TH, which thus up‐regulates dopamine synthesis. Thus, the hypodopaminergic state seen in PKU results not just from reduced precursor penetration into the CNS, but also indirectly through impaired myelination. Supporting this, when PKU mice are treated with a low Phe diet, myelination increases in concert with central DA levels, supporting the strong relationship between these two measures. In humans, this would support the findings that white matter abnormalities on MRI are most significantly related to recent Phe levels (at the time of MRI and in the 12 months prior) and appear to be somewhat reversible (49–51). Additionally, neuroimaging in humans suggests that in addition to hypomyelination in developing axonal tracts, intramyelinic edema may also contribute to the altered T2-weighted signal that is frequently seen in the posterior white matter in PKU patients (52), and changes in posterior white matter on imaging correlate well with serum Phe levels (53).

It is important to note that in Mastrangelo et al.’s retrospective longitudinal long-term study on the outcome of white matter abnormalities in early treated phenylketonuric patients (54), there was no significant association found between cognitive outcome and alterations in white matter structure. However, significant interindividual variability was found, and incongruity was often seen between biochemical control and the trajectory of white matter alterations. About 30% of the variability of white matter alterations remains unexplained by the known quantifiable variables of the disease.

## Psychiatric Disorders

Children, adolescents, and adults with PKU can display disturbed emotional and behavioral functioning with evidence of a heterogeneous range of phenotypes, as summarized in **Table 1**. However, review of the literature continues to demonstrate that the prevalence and severity of problems correlate strongly with the degree and timing of exposure to elevations in blood Phe levels (55). Neuropsychiatric symptoms are likely to be driven by multiple mechanisms, through which hyperPhe impacts brain function, including disruption of amino acid transport across the blood–brain barrier, myelin abnormalities, and reductions in neurotransmitters, as described previously (43).

**Table 1:** Neuropsychiatric symptoms as a function of treatment Initiation.

Untreated	Early-treated children and adolescents	Early-treated adults
Psychotic symptoms	Attentional problems	Depressed mood
Autistic behaviors	School problems	Social isolation/withdrawal
Hyperactivity	Less achievement motivation	Generalized anxiety
Aggression	Low self-esteem	Phobias
Anxiety	Decreased social competence	Social maturity deficits
Depressed mood	Decreased autonomy	Decreased positive emotions
Impaired social skills associated with profound intellectual disability		Low self-esteem Lack of autonomy

Untreated PKU patients have long been known to present with significant neuropsychiatric disturbance, dating back to Følling’s initial descriptions of the disease in 1934, where a spectrum of presentations was noted, ranging from shy and anxious, irritable, agitated and with anger outbursts, through to grossly disturbed, psychotic, and catatonic (56). Further mid-twentieth century literature correlated untreated PKU status with severe behavioral disturbance, largely autistic and psychotic spectrum illnesses, frequently with marked levels of agitation and aggression (57). Literature from that time indicates that this group was particularly challenging to manage in institutions for the intellectually disabled; these patients often display aggression, self-mutilation, impulsivity, and even psychosis (58). In 10 previously undiagnosed and 1 previously diagnosed but untreated individuals, Mazur et al. identified the most frequent behaviors as aggression, affective symptoms including euphoria/elation, as well as agitation and aberrant motor symptoms, but also noted the marked heterogeneity of presentations—including anxiety, delusions and hallucinations, and dysphoria/depression (59).

However with the advent of dietary treatment of PKU, the degree of cognitive and neuropsychiatric impairment markedly reduced in the PKU patient group, with most individuals more assimilated in mainstream educational and vocational settings (60). Given that with strict dietary control, Phe levels in treated patients are usually elevated significantly over Phe levels in non-PKU individuals, it is not unexpected that even with continuous clinical management, psychosocial and/or neuropsychiatric impairments have been observed in clinically treated cohorts (61). Burton et al. demonstrated that >50% of adults with PKU screened positive for the presence of clinically significant psychiatric symptoms, the most common being depression and anxiety (62).

### Depression

Rates of depression are significantly higher among adults with PKU than the general population (63). Depression is reported by up to half of adult patients in survey data (64, 65). A meta-analysis of reported neuropsychiatric complications demonstrated that overall point prevalence rates of depression were 18% in the population with PKU, further subdivided into 12% in the early-treated group, versus 35% in the late-treated (18). This is corroborated in Bilder’s 2017 retrospective cohort study, showing that rates of depression are markedly higher in the overall adult PKU cohort compared to the general population cohort—and this was demonstrated in significantly higher rates of depression in all age groups over the age of 20 years (66). This study of 3,715 individuals with PKU demonstrated depression in 19.5%, which was comparable with depression in a population with diabetes mellitus (21.1%), and exceeding that seen in the in the general population (11.8%).

The underpinning pathophysiology of depression in PKU is likely to be multifaceted. A number of biological processes underpin the development of complex mental illness such as depression, including neurochemical, neurohumoral, and neuroinflammatory processes (67), which are also mediated by psychosocial factors including dietary adherence (68). However, given the key involvement of dopamine, norepinephrine, and serotonin in regulating cognition, emotion, and mood, and the marked elevation of rates of depression in PKU, it is likely that depressive symptoms are strongly associated with hyperPhe-driven low central monoaminergic transmission. This is supported by one study showing that short-term elevations in Phe do result in acutely lowered mood (69). Monoaminergic networks that project to frontal cortical and cortico-limbic regions play an important role in the modulation of mood, and dysfunction in these networks is implicated in the pathophysiology of depression (70, 71). Similarly, antidepressant medications likely act, at least in part if not indirectly, by restoring impaired monoaminergic transmission in these regions (72, 73), particularly through neuroplastic change driven by changes in gene expression and synaptic plasticity (74). Antidepressants may also act by increasing neurogenesis in the dentate gyrus of the hippocampus (75), in addition to other sites, such as the amygdala (76). Adult hippocampal neurogenesis may mediate antidepressant effects *via* its influence on the HPA axis (77).

Unfortunately, the role of antidepressants in the treatment of PKU-associated depression has never been formally studied, so it is not clear if efficacy rates of treatment are comparable to those seen in non-PKU individuals (68), although symptomatic patients may benefit from antidepressant treatment.

### Anxiety

Rates of anxiety disorders are also significantly higher in the overall adult PKU population (15.6%) compared to the general population (9.2%), as evidenced in Bilder’s recent retrospective cohort study (66), and heterogeneity in presentations of anxiety and depression can be largely explained by blood Phe level and PKU treatment (early vs. late/untreated) (18). Anxiety, alongside depression, is one of the most frequently self-reported symptoms in both adult and pediatric PKU patients (15, 64).

A range of anxiety disorders, including generalized anxiety, panic disorder, specific phobias, and obsessive-compulsive disorder, have been associated with low serotonin in the brain (73). Anxiety has been associated with a number of alterations in the serotonergic system including polymorphisms in the serotonin transporter gene (SERT) (78) and also the gene for the CNS-form of tryptophan hydroxylase (TPH2) (79). Given this, it is unsurprising that hyperphenylalaninemia in PKU, and the consequent reduction of central serotonin levels, has been associated with an increased prevalence of anxiety disorders (80). As dietary recommendations in PKU were introduced and, over time, shifted toward a more sustained restriction of Phe intake across the developmental lifespan, symptoms reported by patients also shifted in favor of internalizing symptoms (80). Non-treated children continued to prominently display externalizing symptoms of aggression and agitation in addition to mental retardation, as compared with early-treated children described as exhibiting anxiety, phobias, obsessionality, low self-esteem, and withdrawal (81, 82). Additionally, anxiety measures have been found to correlate with elevations, and significant variability over time, of blood Phe (83). Patients who maintain Phe-restricted diets into adolescence appear less likely to develop an anxiety disorder (15), and those that do have an anxiety disorder are less likely to experience symptoms when they return to dietary treatment (84). Serum Phe levels in PKU have also been shown to correlate to platelet serotonin levels (85), although peripheral are not necessarily a correlate for central serotonin levels (86). An alternate hypothesis for elevated rates of anxiety is that the burden of strict follow-up and dietary treatment constitutes a psychological stressor for children and their families; one study found that patients who adhered to the dietary recommendations during their first decade were more likely to be diagnosed with a psychiatric disorder compared to those patients with poor metabolic control (80); most other studies, however, do suggest that hyperPhe is associated with significant anxiety. For the majority of patients, a reduction in Phe levels often results in a significant attenuation of anxiety symptoms. For a subgroup of patients, particularly those who are unable to effectively lower Phe levels, treatment with serotonergic antidepressants—particularly selective serotonin reuptake inhibitors (SSRIs)—may be beneficial, although data in PKU patients are lacking.

### Psychosis

Psychotic disorders including schizophrenia and bipolar affective disorder, while not the most common neuropsychiatric manifestation of PKU, still occur at higher rates than the general population (66). Early studies of PKU families identified a twice expected rate of psychosis in PKU (87), which has been replicated in more recent retrospective cohort studies (66). Early studies exploring the relationship between Phe, Tyr, and Trp with schizophrenia had mixed results, likely owing to low study numbers and methodological issues. More recent studies in the last 5–10 years have consistently shown a more compelling relationship. Genetic association studies have suggested an association between schizophrenia and PAH polymorphisms, though the functional impact of these genetic changes is not well understood (88, 89). In 2014, Okusaga and colleagues measured plasma Phe in 950 patients with schizophrenia compared with 1,000 controls, and found increased Phe and increased Phe/Tyr ratios, suggesting aberrant PAH function in schizophrenia hypothesized to be as a result of neuroinflammation (90). A small sample of medicated chronic schizophrenic patients was also found to have decreased Phe kinetics as measured by a Phe breath test, as compared to controls, further supporting the role of phenylalanine imbalance in schizophrenia (91). There is significant overlap in the pathophysiology of PKU and schizophrenia, accounting for the increased rates of psychosis seen in this PKU, as well as providing novel avenues for enquiry for the understanding and treatment of schizophrenia.

### Attention Deficit Hyperactivity Disorder

Attention deficit hyperactivity disorder (ADHD) occurs in approximately 3–5% of the population (92). This disorder is characterized by developmentally inappropriate levels of impulsivity, hyperactivity, and/or inattention (93). ADHD is currently categorized into three subtypes with varying rates of prevalence: ADHD-inattentive subtype (approximately 25–30%), the ADHD-hyperactive/impulsive subtype (< 5%), and an ADHD-combined subtype (65–70%) (94). Both PKU and ADHD are highly heritable conditions, though ADHD occurs at approximately twice the rate in those with PKU compared with the general population (66).

Antshel and Waisbren studied 46 children with early- and continuously-treated classical PKU, 15 maternal PKU (mPKU) offspring, and 18 typically developing controls. They demonstrated that both the PKU and mPKU groups had higher ADHD prevalence rates compared to controls (95).

Of note, the inattentive type far exceeded the hyperactive impulsive variant, with the authors postulating that the developmental timing of exposure to elevated levels of Phe may affect the expression of ADHD symptoms. Prenatal exposure is associated with greatly increased hyperactive/impulsive symptoms, whereas exposure at any time affects attention regulation. There is a demonstrated dose-dependent relationship between Phe levels and ADHD symptoms; a greater number of ADHD symptoms were associated with a higher level of Phe exposure (95). Arnold et al.’s study in 2004 mirrored these findings (96).

In examining stimulant use in a group of 38 young people with early- and continuously treated classical PKU (as compared with age-matched type 1 diabetic controls), 50% of youths with PKU reportedly had significant inattentive symptoms, with 26% prescribed a stimulant medication (96). Stimulant use in the diabetic control group was 6.5% comparatively, and population studies suggest the rate of medication use in general ADHD populations is 2–8% (97). Importantly, while the mean Phe levels over 12 months for the PKU sample were significantly associated with inattentive symptoms, medication was reported efficacious in all PKU subjects, without a significant lowering of Phe levels (96).

The link between ADHD and PKU can be at least partially explained by a hypodopaminergic hypothesis (98, 99). In ADHD and PKU affected individuals, this hypodopaminergic state is particularly prominent in the prefrontal cortex and striatum, which rely heavily on optimal neurochemical functioning in order to perform critical roles in attention and behavior (100).

Diamond and colleagues conducted executive function testing in children with PKU longitudinally over 4 years and found that with Phe levels three to five times normal (despite early dietary restriction), they were impaired on tests of working memory and inhibition that were dependent on the functioning of the dorsolateral prefrontal cortex (101). These findings were most correlated to the current Phe level, suggesting that mesocortical system dysfunction underpinned by Phe/Tyr imbalance and subsequently reduced dopamine dysfunction was the underlying issue (101). Projections to prefrontal areas are thought to be more vulnerable to this hypodopaminergic state owing to their higher turnover of dopamine (102). A number of studies have linked dopamine to the neurobiology of ADHD, including those showing a link with dopamine system genes (103), overexpression of the dopamine transporter (104), and the pharmacological basis for effective treatments (105).

Given the particular sensitivity of the prefrontal cortex to low levels of dopamine, it is not surprising that this area is disproportionally affected by the sequelae of even mild elevations of Phe (102), and this provides a likely mechanism for the increased rates of ADHD in PKU. There are limited studies of the link between the two disorders, despite the link in neurobiological mechanisms.

The PKU ASCEND study was conducted to evaluate the therapeutic effects of sapropterin versus placebo on ADHD and executive functioning in PKU patients, and undertaken in individuals who had a demonstrated therapeutic blood Phe response to sapropterin therapy. Burton et al. conducted a randomized control trial including 206 children and adults with PKU (mean age = 20 years), of whom 118 responded to sapropterin therapy (106). There were 38 individuals that had sapropterin responsive PKU and ADHD symptoms at baseline. Primary endpoints were changed on ADHD Rating Scale (ADHD RS—child parental report and ADHD-ASRS—adult self-report versions), the Clinical Global Impression of Improvement (CGI-I), the Clinical Global Impression of Severity (CGI-S), and the Behavioural Rating Inventory of Executive Function (BRIEF). Sapropterin therapy was found to reduce inattentive ADHD symptoms in the first 4 weeks of treatment, and throughout the 26 weeks of treatment, these improvements were maintained. There were no changes noted between groups on the hyperactivity and impulsivity measures from the ADHD RS/ASRS. One third of the 118 individuals were found to have ADHD inattentive symptoms, similar to the findings of Antshel et al. (95).

## Cognition in PKU

### Cognitive Functioning in Early Treated Children With PKU

The historical literature on neurocognitive outcomes in children with early treated PKU is vast, and a number of meta-analyses have been conducted to try and overcome some of the methodological challenges, including small sample sizes, heterogeneous samples, small effect sizes, and large variability in outcomes. Mild to moderate deficits have been reported in a range of domains, including general intelligence (107, 108), attention (109–112), processing speed (112–114), working memory (115, 116), new learning and memory (112), motor skills and co-ordination (117), and executive functioning (112). Aspects of executive functioning are particularly sensitive to high Phe levels, and deficits have been reported in a variety of executive subdomains, including inhibitory control (101, 114), conceptual reasoning (118), planning (118–120), mental flexibility (114), and organizational strategy (115). Academic achievement differences have also been observed in some studies (112). White matter pathology in PKU is diffuse, and as a consequence, multiple pathways may be compromised, causing a broad array of neuropsychological difficulties in the PKU population (121).

DeRoche and Welsh (122) conducted a meta-analysis looking at 25 years of neurocognitive outcomes in children and adolescents with PKU, spanning from 1980 to 2004 including 33 studies (122). Effect sizes for intelligence (in comparison to unrelated controls) was small to moderate (0.20 to 0.42) with no significant heterogeneity among outcomes. The effect sizes for executive functioning (and its component processes) were in the “moderate to large” range including working memory (0.59), planning (0.51), inhibition (0.78), and flexibility (1.15). Significant heterogeneity among effect sizes was noted, though all domains of executive functioning were found to be significant (p < 0.001). DeRoche and Welsh concluded that individuals with early and continuous treatment will likely have IQ scores in the average range, albeit possibly lower than genetically related controls. However, they may experience significant impairments in executive functioning, including flexibility of thinking, inhibition, working memory, and planning (122).

As early treated children and adolescents with PKU commonly demonstrate executive difficulties on neuropsychological examination, it is important for parents, teachers, and the individuals themselves to be mindful of potential challenges in academic and social situations. As children progress through their schooling, they face many novel and challenging situations, demanding of executive skills and goal-directed behavior. Self-regulation and cognitive control are related to successful school achievement and social adjustment (123). Therefore, individuals with PKU may require additional educational support and counselling to help compensate for executive difficulties (122).

### Cognitive Effects of Phe Variability in Children With PKU

Relatively few studies have examined the neuropsychological outcomes in mild hyperphenylalaninemia, in part due to a lack of consensus regarding the definition of mHPA (61). However as blood Phe concentrations can increase with age, children should be monitored during the first year of life at a minimum (124, 125). The evidence regarding cognitive outcomes in children with blood Phe levels just under 600μmol/L is inconsistent, with small sample sizes and methodological flaws (126, 127). A number of studies have focused solely on IQ assessment, rather than a comprehensive review of cognitive abilities, including executive functioning. The existing literature indicates that children with mHPA generally do not have intellectual impairment, but usually perform between those of individuals with phenylketonuria and those of comparison groups on broader neuropsychological measures (126, 127). However there is evidence that even modest elevations in Phe can affect selected cognitive functions, including attention, working memory and executive functioning (61, 126, 127). The complete European guidelines for PKU diagnosis and treatment err on the side of caution and recommend that children with blood Phe levels between 360μmol/L and 600μmol/L should be treated during the first 12 years of life to optimize cognitive functioning (17).

### Cognitive Functioning in Early Treated Adults with PKU

There has been considerable research into the cognitive functioning of children with PKU but less so in adult populations (128, 129). For the majority of adults that commenced treatment shortly after birth, individuals generally fall within the normal range of general cognitive ability, have professional and educational achievements similar to their non-PKU siblings, and are able to live independent and productive lives (17, 128). However, there are some individuals that continue to demonstrate neuropsychological, social, and behavioral difficulties throughout their adult lives. These challenges can impact on education and training, employment, relationships, emotional wellbeing, and quality of life (1, 130, 131).

Palermo et al., (132) undertook comprehensive neuropsychological assessment on 37 early treated adults with PKU (AwPKU) with good dietary control (average 432 µmol/L childhood and <850 µmol/L in adulthood) and 30 controls (132). They found that only 5.4% of the PKU participants showed severe impairment (> 2 SD below the mean); however, a much greater number showed an abnormal cognitive profile in terms of proportion of impaired measures (46%). Overall, a quarter of the sample showed clear cognitive impairment in terms of average performance and cognitive profile, whereas 38% performed as well as controls with z scores always within 0.5 of the mean across all assessed domains.

When comparing AwPKU and controls, the largest difference (Cohen’s D) was found for planning and switching (Tower of Hanoi, Wisconsin Card Sorting Test, Semantic Fluency), verbal reasoning (WASI Similarities and Vocabulary), short-term memory (Digit Span and Non-Word Repetition), and sustained attention (Rapid Visual Information Processing). Visuo-motor coordination (Grooved Pegboard and Digit Symbol) was reduced in comparison to controls but was thought to reflect general cognitive slowing rather than a specific difficulty with motor coordination or peripheral motor speed. Reading speed was slower than controls but was accurate. Spelling was intact, as was picture naming. Memory and learning were preserved [Rey Auditory Verbal Learning Test (RAVLT), Paired Associate Learning (PAL)].

Hofman et al. (129) reviewed 22 peer-reviewed publications, reporting on the outcomes of 16 studies of cognition in early treated PKU (129). Across studies, the most consistent findings were deficits in motor skills, working memory, and vigilance. However, impairments in other cognitive domains were less consistently observed. The relationship between Phe levels and cognition across the lifespan was variable, with no definitive linear pattern of association found. This was thought to reflect a number of core challenges in studying cognition in PKU. These included high heterogeneity in the nature of study samples, resulting in large variability in phenylalanine levels, as well as wide variation in the type and sensitivity of neuropsychological measures used to assess cognitive functioning. Study samples were often small, with cohorts exhibiting different levels of disease severity, age ranges, and socioeconomic status. No standard PKU core neuropsychological battery had been uniformly used. Hofman et al. concluded that the long-term cognitive outcome of continuous early management of PKU remained unclear.

Weglage et al. (133) conducted a longitudinal neuropsychological and neurological study of 57 early-treated classical PKU adults aged between 19 and 41 years and 46 controls over a 5-year period (133). PKU participants and controls were assessed on IQ, attention, and information-processing abilities. Magnetic resonance imaging (MRI) of the brain was performed in all patients. Neuropsychological assessments and MRI were repeated at a 5-year follow-up. In the 5-year interval, there was no change in processing speed or attentional measures. At both assessment times, IQ scores were significantly lower in PKU individuals as compared to controls. Older adult patients (> 32 years) showed poorer attention and information processing at both timepoints compared to young adult patients (aged <32 years) and controls. Intellectual quotients, attention, and information processing showed no correlation to imaging results; however, they were significantly correlated to blood phenylalanine (Phe) levels throughout childhood and adolescence. Phe levels had also been higher in the adolescent years of older adult patients, suggestive of an early relaxation of diet recommended when the older patients were adolescents. Weglage’s results indicated a benefit of dietary control during adolescence in PKU (133).

### Cognitive Outcomes in Late Treatment or Untreated PKU

Untreated PKU is characterized by motor deficits, intellectual disability, microcephaly, autism, seizures, developmental problems, behavioral issues, and a range of psychiatric symptoms (17). Failure to implement treatment in the neonatal period causes substantial lifelong disability through the toxic effects of excess Phe exposure to the brain, in particular to myelin and dendritic projections during critical postnatal periods of neuronal development (18). Brain MRI and histopathology of individuals with late- or never-treated PKU demonstrate diffuse cortical atrophy, hypomyelination, white matter vacuolization, and astrocytic gliosis (47, 134).

Gonzalez et al. (135) conducted a retrospective study of 121 PKU individuals (mean age = 16, range 1 month to 46 years) diagnosed and treated from 1985 to 2010 (135). The aim was to investigate the relationship between neurological complications and behavioral problems, age at diagnosis and dietary control among a follow-up group (135). Of these, 76% were diagnosed through neonatal screening. There were 12.4% with mild PKU, 19% moderate PKU, and 68.6% classic PKU. Eighty-eight percent of patients were treated with a protein-restricted diet, and the remainder with BH4. Almost all (97.7%) of the early-diagnosed patients had normal IQ, while 46.3% of late diagnosed patients had intellectual disability, 28.5% were borderline, and 25% had normal IQ.

In early-diagnosed patients, there was a significantly negative correlation between IQ and the index of dietary control during the first 6 years of life and that of the immediately preceding year. The proportion of patients with late diagnosis and neurological and behavioral problems was significantly higher than that of those diagnosed early. In addition, the proportion of early-diagnosed patients with behavioral and neurological problems who had good, intermediate, or poor dietary control during the first 6 years of life (and the immediate-past year) also differed significantly (135).

The severe cognitive impairments seen in untreated PKU can be partially reversed with dietary treatment in many individuals, and the prompt initiation of treatment following newborn metabolic screening remains essential for the prevention of disability and optimal neurodevelopment.

### Intelligence in PKU

PKU results, in untreated patients, a profound intellectual disability and more subtle cognitive deficits in individuals who were treated early and continuously. The assessment of intellectual functioning in PKU has been an important target outcome variable since the implementation of neonatal PKU screening programs in the 1960s (108, 136). Research on intellectual functioning in individuals with PKU has played a significant role in guiding treatment recommendations and mproving outcomes (136).

Brumm and Grant (136) conducted a literature review examining the relationship between intellectual outcome and treatment parameters including initiation of treatment, duration of treatment, and blood phenylalanine (Phe) levels from infancy through adulthood. While current PKU treatment practices have eliminated severe neurological and cognitive impairment, evidence suggests that intellectual functioning, although typically within the average range when PKU is treated early and continuously, may not be maximized under the current definition of well-controlled PKU, which is based on blood Phe levels (136).

Two prior meta-analyses examining blood Phe concentrations and intellectual functioning in pediatric populations found a strong inverse relationship between historical blood Phe measurements and IQ (137, 138). Future research assessing intellectual and neurocognitive outcome in PKU should enhance the development of new treatment strategies.

### Executive Functioning in PKU

Executive function is an umbrella term that refers to cognitive processes that are necessary for purposeful, future orientated behavior. Metaphorically speaking, the executive functions are the brain’s chief executive officer. These processes are necessary to plan and complete tasks in spite of potential distracting or irrelevant information (139).

Executive functions include cognitive processes such as regulation of attention, inhibition of inappropriate responses, coordination of information in working memory, and cognitive flexibility. Higher-order executive functions require the simultaneous use of multiple basic executive functions and include planning, reasoning, and problem-solving (140).

Reviews of executive functions have found that the most consistent phenylalanine-related impairments have been observed in working memory, sustained attention, and inhibitory control (65, 129, 141). There have been several investigations to try and establish a theoretical model of executive functioning deficit in individuals with PKU, which has resulted in detailed analysis of various aspects of executive functioning (142). Part of the difficulty in the assessment of executive functioning in PKU populations has been the lack of consistent use of valid and sensitive tools that are suitable for both children and adults (141). Another challenge has been that many traditional “executive functioning” tasks do not solely measure one specific cognitive process. As an example, an executive test such as Trails B indexes both speed and mental switching (143).

Traditional tasks that have been used to assess executive functioning in PKU are the Wisconsin Card Sorting Test (WCST) and Brixton Spatial Anticipation Test (rule detection), Tower of London and Tower of Hanoi (planning and problem solving), Rey Complex Figure Test (visuo-spatial organization), Trails A and B and the Contingency Naming Test (mental flexibility and switching), the Controlled Oral Word Association Test/Verbal Fluency (verbal generativity and planned searching), and the Go-No Go Task, Stroop Test, and Haylings Sentence Completion Test (Inhibitory Control) (113, 122, 129, 142). Computerized test batteries have also been used to assess executive skills in PKU populations, including the Amsterdam Neuropsychological Test and the Cambridge Neuropsychological Test Automated Battery (CANTAB) (65, 69, 141). Studies have demonstrated significant variability in their findings, in part due to the methodological challenges in conducting research in rare and diverse PKU populations. In the future, it will be important for a consistent and sensitive core neuropsychological battery to be implemented across international PKU centers, to try and standardize consensus regarding cognitive treatment outcomes (17, 129).

### Cognition in Offspring of Mothers With PKU

High Phe blood levels during pregnancy have a known teratogenic effect on the developing fetus, resulting in growth retardation, microcephaly, intellectual disability, and birth defects (144). There have been several studies that have assessed the cognitive outcomes of children born to mothers with PKU (144-146). A summary of the literature suggests that children who are born to mothers with PKU who have attained metabolic control before or very early in pregnancy seem to have a normal developmental trajectory. However, a delay in attainment of maternal metabolic control is associated with declines in offspring developmental outcome, including lower IQ and higher rates of externalizing behavioral difficulties (145).

Waisbren and Azen (145) conducted a prospective longitudinal study that assessed cognitive and behavioral outcomes in treated (mPKU) offspring (145). Two hundred and twenty-eight children who were born to mothers with treated PKU or untreated mild hyperphenylalaninemia were compared with 70 control subjects at 7 years of age. They found that the offspring cognitive outcome negatively correlated with the number of gestational weeks that elapsed until maternal metabolic control was achieved (r = _-_0.61). There was an increased risk of low IQ in PKU offspring if the mother came from a lower SES background and was also unable to provide a stimulating early home environment (145). The postnatal environment also significantly affected outcome. Interventions to improve dietary compliance before and throughout pregnancy may reduce the risks associated with mPKU (17).

### Social Cognition and PKU

Social cognition is a domain of cognition involving all mental processes that underlie social interactions, and encompasses the ability to perceive, interpret, and then respond appropriately to social cues. Some basic social cognitive skills include face and emotion recognition and theory-of-mind (the capacity to attribute and understand feelings, thoughts, and intentions to/of others) (147). While there has been comprehensive investigation into the role of executive functions in PKU, there has only been one study to date that has assessed social-cognitive abilities (148). Deficits in social cognitive abilities are consistently reported in individuals with executive dysfunction, because of shared underlying neurobiology and neuroanatomy (149–151). In other disorders affecting the CNS, impairments in executive function have been correlated with deficits in communication skills and social relationships (152). High Phe levels may in some cases result in irritability, impacting on adaptive social skills.

(148) investigated whether early treated PKU patients have specific Phe-related problems with respect to social-cognitive functioning and social skills (148). Ninety-five PKU patients (mean age 21.6 ± 10.2 years) and 95 healthy controls (mean age 19.6 ± 8.7 years) were compared on performance of computerized and paper-and-pencil tasks measuring social-cognitive abilities and on parent and self-reported social skills. Early treated AwPKU performed worse than controls on all four tasks included; however, when age was controlled for, impairments were only observed on two tasks. In addition, comparisons were made between patients using tetrahydrobiopterin (BH4, n = 30) and patients who were not. PKU patients demonstrated poorer social cognition and had poorer social skills than controls, regardless of general cognitive abilities. The quality of patients’ social cognition was inversely related to recent Phe levels, and to levels between 8 and 12 years, for PKU adolescents. The quality of social skills was also inversely related to lifetime phenylalanine levels in adult patients, and more specifically to Phe levels up to 12 years of age. No differences with respect to social outcome measures were observed between the BH4 and non-BH4 groups. Jahja and colleagues concluded that PKU patients have difficulties with social cognition and social skills that appear to be strongly related to Phe levels. Impairments in functioning seem to be more evident among adolescents and adults with PKU, with high Phe levels during childhood and early adolescence (presumably during critical periods of development of frontal circuits subserving social cognitive functioning) seem to be of greater influence than current and recent Phe levels in PKU individuals (148).

### Neuropsychological Mechanisms in PKU

While there has been no consensus agreement about the exact mechanism/s for cognitive changes in PKU, the general belief has been that the deficits are related to individuals’ Phe levels at several stages throughout life (153). These include concurrent Phe levels, lifetime Phe levels, variation in Phe levels, and altered Phe/Tyrosine ratio. Two theories have been postulated regarding the mechanism of action of the disrupted Phe metabolism in individuals with PKU. The first theory suggests that because Phe competes with other LNAAs (tryptophan and tyrosine) for transport across the blood–brain barrier, high levels of Phe saturate the LNAA transporters. This leads to PKU individuals presenting with lower brain concentrations of other LNAA and important neurotransmitters such as dopamine, norepinephrine, and serotonin (153), which are known to be important in cognitive functioning (154). The second theory suggests that high brain Phe concentrations cause neurotoxicity. This interferes with cerebral protein synthesis, increases myelin turnover, and inhibits neurotransmitter synthesis (43).

One of the primary cognitive changes that is consistently seen in the AwPKU literature is a reduction in speed of processing. This is reflective of cognitive slowing, rather than a peripheral reduction in motor speed (132). Information processing speed relies on the speed with which action potentials are able to travel along the long myelinated axons. If myelination of these axons was damaged or incomplete, there would be a reduction in processing speed. Previous studies have found that slowing occurs at a cognitive level, consistent with the apparently toxic effects of hyperPhe on oligodendrocytes (132, 155, 156), but not on the peripheral nervous system’s Schwann cells (157).

### Neuropsychological Assessment Recommendations

There is currently no consensus regarding a standardized PKU neuropsychological battery for children or adults. The European guidelines suggest that neuropsychological assessment should occur on an “as needed” basis in childhood, with routine evaluations at 12 and 18 years of age. This correlates with changes in treatment targets for blood Phe and life changes including school transitions, living situation, and transfer to adult clinics and brain development (see **Table 2**).

**Table 2:** Neuropsychological assessment recommendations.

Neuropsychological assessments should be conducted at 12 and 18 years in all patients
If any of the stated risk factors applies, perform (additional) neuropsychological assessment: Non-optimal metabolic control; <50% of the Phe levels are out of target range over a period of 6–12 months (depending on age <12 or >12 years) Problems at school or work Concerns of parents/caregivers/family/teachers Concern of PKU patient Concern of metabolic team
**Cognitive Domains for Inclusion**
Estimation of intelligence Academic achievement Immediate, divided, and sustained attention Speed of processing Visuo-spatial functioning Verbal new learning and memory Expressive and receptive language Motor speed and coordination Executive functions: Working memory Inhibitory control/self-monitoring Planning and organization Cognitive flexibility/shifting Verbal fluency Social cognition Psychiatric screening Psychosocial adjustment/quality of life Behavioral rating scales

**Table 2** is adapted and expanded from Statement #24, European Guidelines for Diagnosis and Treatment of PKU (17).

When conducting a neuropsychological assessment in individuals with PKU, it is important for clinicians to conduct a comprehensive examination encompassing all cognitive domains. If time is limited, the domains most likely to be susceptible to high Phe levels are attention, working memory, motor control, complex speed of processing, and executive functioning (17,129, 141). Intellectual functioning as a stand-alone outcome measure has been found to be less sensitive than the above-mentioned cognitive abilities (141). While memory retention is often sound, attentional difficulties can affect registration and encoding of new information and should be assessed.

A thorough clinical history should be obtained prior to an assessment being conducted, to help guide test selection, with a particular focus on cognitive strengths and weaknesses, social cognition, psychiatric symptoms, psychosocial adjustment, quality of life, and behavioral difficulties (17,158). As there is emerging evidence that social cognition skills (facial and emotional recognition and theory of mind) and emotional and behavioral regulation are affected in individuals with PKU, it is important to consider assessment of these domains as part of a comprehensive neuropsychological examination (148). A brief psychiatric history should also be obtained, as conditions such as depression and anxiety can negatively impact on cognitive test performance, particularly on measures of attention and speed. Mood and anxiety symptoms can be treated, which may lead to an improvement in cognitive functioning. By conducting a comprehensive neuropsychological examination, cognitive, psychological, and social cognition interventions can be individually tailored to best assist individuals with PKU to achieve their highest potential and enhance quality of life.

## Case Vignettes

### Case 1

**Figure 2:**
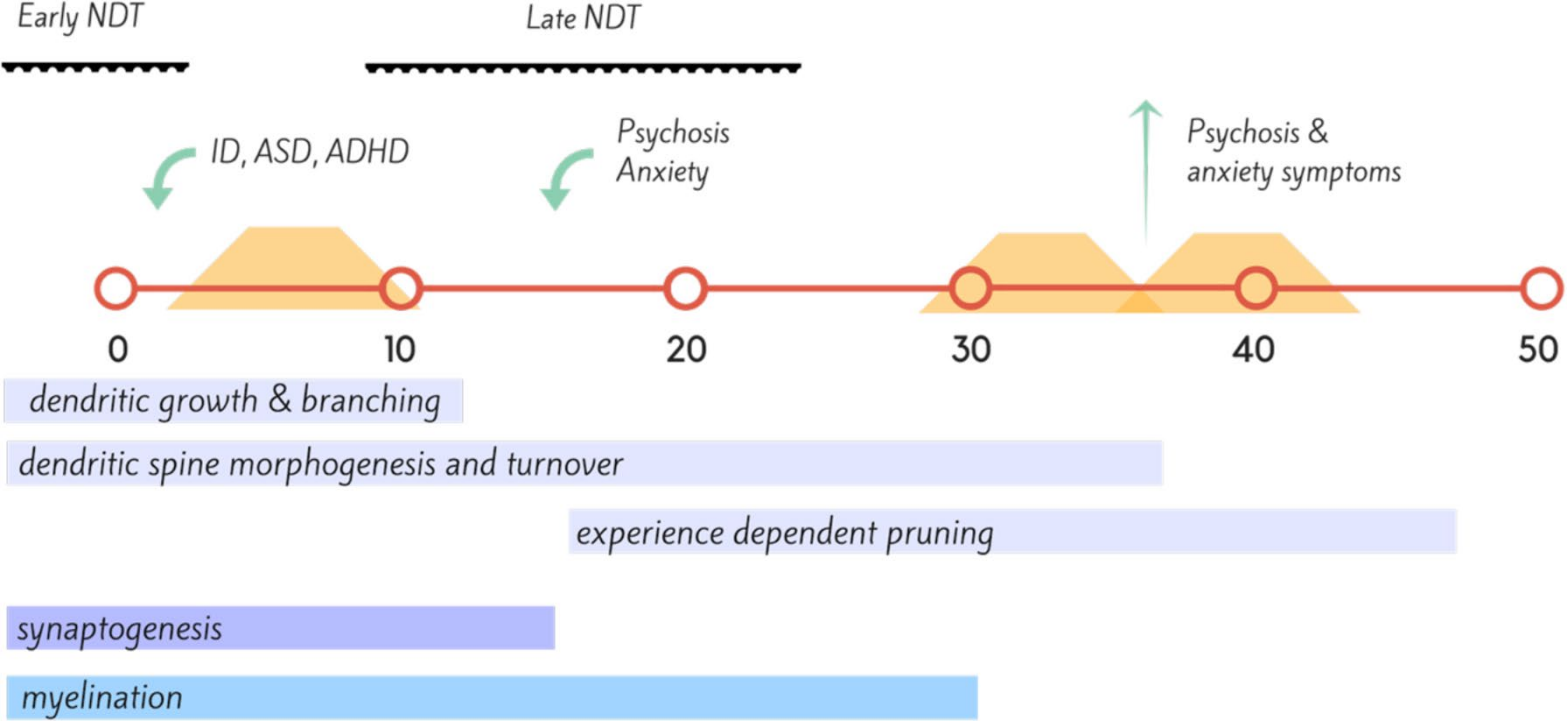
Timeline (in years) of proposed neural development, onset of neuropsychiatric disorder (green) and dietary coverage (orange) for Case Vignette 1. This case highlights the importance of neuronal development across the lifespan, with insult during the early neurodevelopmental (NDT) stages resulting in intellectual disability (ID), autism spectrum disorder (ASD), and attention deficit hyperactivity disorder (ADHD); while insult during the later stages of neurodevelopment, during adolescence with disruption of synaptic pruning leading to disorders such as bipolar disorder and in this instance, schizophrenia (159, 160).

Ms T was a 43-year-old single woman who was not diagnosed with PKU until after 12 months of age and only started dietary treatment at 18 months of age. Prior to this period, she had significant irritability, childhood anxiety, delayed milestones, and failure to thrive and progress (**Figure 2**). She continued dietary treatment until the age of 9, at which time the family was told to stop dietary management. Ms T’s IQ was in the intellectually disabled range (<70). She did not resume diet until 30 years of age. She presented with attentional deficits, hyperactivity, and social cognition issues throughout childhood, and then developed a psychotic illness in her late teens. This remained largely treatment refractory, and during periods of poor dietary control (Phe >1500), she suffered from poor frustration tolerance and impulse control, anxiety, and worsened chronic hallucinations. When dietary control was good (Phe 400–600), psychotic symptoms were significantly attenuated, anxiety was minimal, and impulse control returned to normal.

### Case 2

Mr C was a 47-year-old married engineer and father of two, who was diagnosed at birth on bloodspot screening. His dietary control was “strict” until age 13, “relaxed” until age 17, then ceased dietary control altogether. At times of increased protein intake, he noted increased mood lability, irritability, and anxiety, with inattention and a sense of “cloudy” thinking; this occurred with Phe levels at 1,200–1,400. His anxiety was generalized, with occasional panic episodes and periods of low mood with mild neurovegetative disturbance. With improved dietary control (Phe < 700) he noted that his mood lifted, anxiety levels reduced, and sleep improved significantly. After 12 months of dietary control, anxiety levels had improved markedly, and his mood was persistently euthymic.

Mr C underwent comprehensive neuropsychological assessment prior to commencing a low Phe diet (baseline) and after 12 months of good dietary control. At baseline, Mr C was estimated to be of “Average–High Average” intelligence. He performed in keeping with these estimates on all tasks, with the exception of verbal new learning and memory, where he was in the *Borderline Impaired* range. Mr C reported that he found it challenging to attend to the verbal information being presented.

After 18 months on diet, Mr C demonstrated statistically significant improvements in the domains of processing speed (from 34 percentile at baseline to 77 percentile on-diet) and new learning and memory (from 7 percentile at baseline to 50 percentile on diet). A number of executive functioning measures also improved, although the improvements were mild and not statistically significant. He reported being able to think more clearly and quickly, with improved concentration.

### Case 3

**Figure 3:**
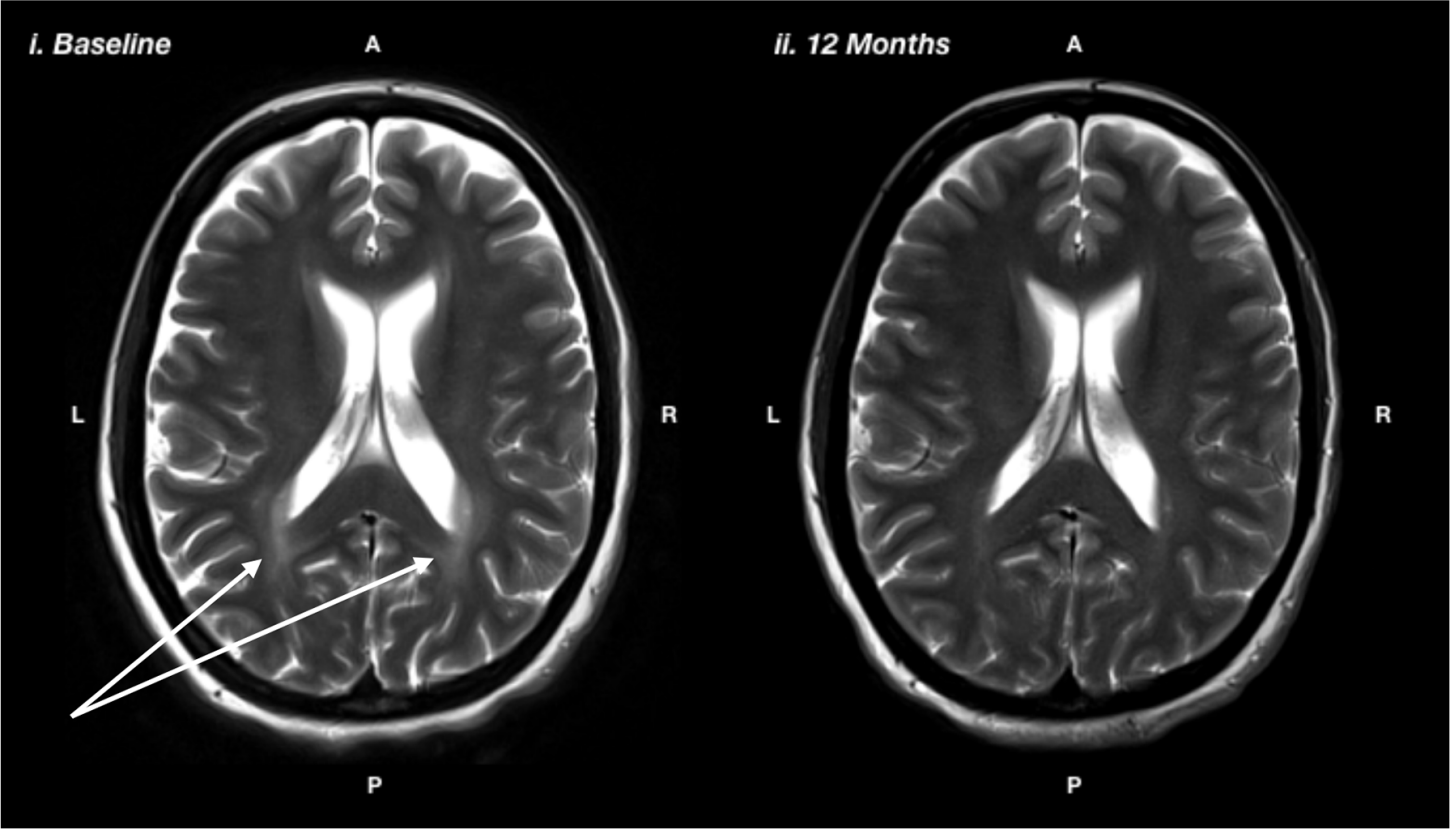
T2-weighted magnetic resonance imaging showing i) baseline: prior to the recommencing diet, and ii) 12 months after recommencing diet, showing reduction in white matter hyperintensity, particularly in the posterior regions, as indicated by the white arrows.

Ms N was a 36 year-old married lady who was diagnosed at birth on bloodspot screening. She adhered to a low Phe diet for the first 7 years of her life then ceased dietary control. She recommenced on diet at age 35 due to symptomatic PKU affecting her cognitive functioning and mental health. Ms N had a long history of mental health difficulties starting in childhood. She had difficulties regulating her mood and had episodes of depression. She had her first panic attack in her mid-teens, along with generalized anxiety symptoms and infrequent self-harming behaviors. She had been under psychiatric care since her teenage years and had several psychiatric admissions. She had worked in a variety of semi-skilled roles in a part-time capacity, but had found it difficult to maintain employment due to fluctuating mental health. She saw a psychologist on a regular basis and had been on pharmacological treatment with SSRIs since her early 30s, with some improvement. She reported significant cognitive limitations, including difficulties with attention and concentration, memory, planning, and organization and slowed mental processing. These difficulties had affected her ability to maintain productive employment, leading to feelings of inferiority and low self-esteem. Baseline neuropsychological assessment prior to resuming a low Phe diet revealed that Ms N was of average intelligence. However, she demonstrated moderate impairments on tasks of divided attention and psychomotor speed and severe impairments on task of planning, organization, and self-monitoring. After 12 months of good dietary control (reducing Phe from ∼700 to <300), Ms N had made statistically significant improvements on tasks of psychomotor speed (from 9 percentile at baseline to 63 percentile on diet), planning and organization (from <0.1 percentile at baseline to 77 percentile on diet), divided attention (from 9 percentile at baseline to 50 percentile on diet), and self-monitoring (from 1 percentile on diet to 37 percentile on diet). She also showed a significant regression in white matter lesions (**Figure 3**). Her depression and anxiety symptoms also improved; however, they did not fully resolve and she benefitted from the introduction of escitalopram, initially at 20 mg but ultimately required a dose escalation to 40 mg.

### Treatment Considerations: Current and Future

Though rare, PKU is an important cause of preventable neurodevelopmental disability that requires lifelong commitment to Phe restriction. The literature supports treatment being initiated as early as possible, even before 10 days, in order to minimize neurological damage. Every 4-week delay in starting treatment has been shown to cause a decline of IQ score by approximately 4 points (1).

There is consensus in the literature that patients with untreated blood Phe concentrations > 600 μmol/L should be treated, and that patients with untreated blood Phe levels < 360 μmol/L should remain untreated, as this is not considered to be indicative of disease (138). What is less clear is treatment in the group of patients with PKU whose levels are in the 360 and 600 μmol/L range. The most reliable literature suggests they should be treated during the first 12 years of age to prevent cognitive function impairment in PKU (161). Because of the consequences of elevated blood Phe levels, guidelines from the American College of Medical Genetics and Genomics (ACMG) recommend lifelong treatment of PKU, with the primary goal of therapy to lower blood Phe to the range of 120 to 360 μmol/L (16).

Dietary treatment is comprised of three aspects: natural protein restriction, Phe-free- L-amino acid supplements, and low protein food (17). Though central to the treatment, dietary modification remains very challenging for patients and their families, due to cost and impact on quality of life, and despite optimal dietary treatment, outcomes remain suboptimal, hence the ongoing exploration into alternate treatments.

A patient’s Phe tolerance will vary over their lifetime, depending on many factors such as the stage of growth and development and net protein catabolism-synthesis ration, target Phe levels, severity of illness, energy intake, compliance with diet and supplementation, as well as use of adjunctive treatments such as BH4 (162, 163). Striking a balance between adequate protein intake and maintaining acceptable Phe levels needs to be individualized to each patient (17).

### Amino Acid Supplementation

Phe is an essential amino acid for protein synthesis both in childhood and adulthood and is gained through dietary protein intake (164). Traditional treatment of PKU has been to restrict overall dietary protein intake (165). However, as the understanding of PKU neuropathophysiology has unfolded, it has become clear that just keeping Phe levels low does not constitute optimal treatment.

A mainstay of dietary treatment is supplementation with Phe-free L-amino acid supplements. The 2017 van Wegberg et al. European Guidelines on Phenylketonuria Diagnosis and Treatment state that supplementation should be evenly administered throughout the day in at least three equal portions to minimize losses of L-amino acids due to oxidation, and to help minimize fluctuations in blood Phe concentrations over a 24-h period (17, 166).

Oral supplementation with LNAAs has been proven to be helpful in reducing brain Phe concentrations *via* competitive binding at LAT1, which improves neuropsychological functioning (167, 168). LNAA supplementation either alone or in combination with a low-Phe diet has been shown to improve health outcome for individuals unable to follow the low-Phe diet. However, long-term outcome studies assessing efficacy and safety of LNAA supplementation are needed (169).

Promising developments include van Vliet et al.’s studies on mice showing that LNAA supplementation without dietary phenylalanine restriction improves brain biochemistry through all three hypothesized biochemical mechanisms: namely, the normalization of brain phenylalanine, non-phenylalanine LNAA, and the concentration of monoaminergic neurotransmitters. They demonstrated that utilizing LNAAs with a strong affinity for the LNAA transporter type 1, such as Leu and Ile, is most efficacious in reducing Brain Phe concentrations. Additionally, Tyr and Trp in conjunction serve to optimally ameliorate brain monoaminergic neurotransmitter concentrations (170). They also compared several LNAA supplements with a very phenylalanine-restricted diet on measures of brain monoamine and amino acid concentrations in adult C57Bl/6 Pah-enu2 mice, and the results indicated that the supplementation of the eight LNAAs was as effective as the highly Phe-restricted diet in restoring CNS monoamines, even while brain and plasma phenylalanine concentrations remained highly elevated (171).

### Advances in Dietary Supplementation

Dietary modification has continued to evolve to ensure that foods are affordable, palatable, and supplemented with micronutrients otherwise lacking in a low-Phe diet. One such advancement is the utility of glycomacropeptide (GMP), an intact protein source derived from cheese whey that improves Phe utilization and protein retention (172). Compliance has been shown to be improved with GMP as compared with traditional amino acid foods due to greater acceptability and fewer gastrointestinal side effects (173).

Other supplementation changes include improved caloric content, addition of taurine and other micronutrients, and addition of long-chain polyunsaturated fats (LCPUFAs) (171).

### Long-Chain Polyunsaturated Fatty Acids

LCPUFAs have been found to be decreased in systematic review and meta-analysis of studies in PKU, likely secondary to dietary restriction and possibly metabolic changes, though the latter is only partially understood (174, 175). The addition of omega-3 docosahexaenoic acid (DHA) to the diet of children with PKU has been shown to increase central nervous system processing speed (as measured by visual evoked potentials) as well as motor and coordination skills, suggesting DHA is an essential part of the treatment of PKU (176).

### Tetrahydropterin as Enzyme Enhancement Therapy for PKU

Tetrahydropterin acts as a molecular chaperone in PKU patients and promotes correct folding and stability of the PAH enzyme, thus acting as a critical co-factor in a normal PAH system (177). A subset of patients with PKU benefit from adjunctive treatment with pharmacological doses of tetrahydropterin (BH4) or sapropterin hydrochloride (178). Robust evidence from two systematic reviews demonstrates that BH4 is effective in reducing blood Phe concentrations and in increasing the Phe “tolerance” in BH4-responsive PKU patients (179, 180). Long-term treatment with sapropterin of such responsive patients with PKU also appears to improve Phe tolerance and may allow patients to discontinue highly restrictive diets (181).

However, not all patients benefit, and those with Phe levels > 360 µmol/L need to be tested for responsiveness. This requires multiple levels to be taken pre and post the initiation of BH4, and a decrease in blood Phe of 30% or more from baseline indicates response to sapropterin therapy (130). Patients with high residual activity of the PAH enzyme have a greater probability of BH4 response, but a minority of patients with classical PKU have been shown to benefit from BH4 treatment (182, 183). Understandably, BH4 responsive patients have been shown to have a higher natural protein tolerance (182, 183).

There is ongoing research required to establish if other molecules in addition to tetrahydrobiopterin may act as chaperones to assist in the folding of PAH; however, there are some promising studies including one by Pey et al, which performed a high-throughput ligand screening of over 1,000 pharmacological agents, identifying four compounds that enhanced the stability of PAH activity (184). Many patients who have little or no residual PAH activity do not respond to BH4. Studies have identified various figures from 20% to 50% of patients responding in the studies to combination sapropterin and dietary Phe restriction (185, 186).

### Enzyme Therapy

Pegvaliase, PEGylated recombinant *Anabaena variabilis* phenylalanine ammonia lyase (PAL), converts Phe to transcinnamic acid and ammonia, and is now FDA-approved as an enzyme substitution therapy to lower blood Phe in adults with PKU (187). As per Thomas et al., Pegvaliase has now been investigated in a single Phase 1 clinical trial, four Phase 2 clinical trials, and two Phase 3 clinical trials, PRISM-1 and PRISM-2, the results of which are now reported (188–190). These studies have evaluated over 350 adults with PKU being treated with Pegvaliase.

The PRISM clinical program exemplified that with a manageable self-administration program, patients with PKU were able to achieve sustained reductions in blood Phe concentration, that were associated with sustained improvements in associated neuropsychiatric symptoms (188). Evidence from Vockley et al. is demonstrating that long-term treatment with Pegvaliase showed consistent and clinically meaningful improvements across all inattention and mood outcomes evaluated (191), representing an exciting new treatment pathway (192).

### Individual Variation and Compliance

Interestingly, studies indicate that while there are patients with PKU suffering from all the sequelae of non-treatment, or poor adherence to treatment, so too there is likely a subset of patients who are over-restricting their protein consumption. Blood levels of Phe do not necessarily fluctuate with small changes in dietary intake (162, 193). For those with either late diagnosis or no previous treatment, there is a growing body of evidence that introduction of the low Phe diet and adjunctive treatments will benefit patients and can reverse some of the IQ loss in reported cases, particularly in the younger age group 4 to 6 years old (194). Concerningly, there are high rates of attrition from services, with some services reporting that a majority of adults with PKU were lost to follow-up and, without the support of specialist services, are likely to have suboptimal metabolic control (195, 196).

### The Role of Sleep

Accumulating evidence increasingly indicates that improving quality of sleep is an important target for intervention in optimizing the cognitive and psychiatric state of those with PKU. Bruinenberg et al. explored the link between PKU and sleep disturbance in men and mice in their 2017 study and asserted that PKU patients demonstrated more sleep disorders, a reduced sleep quality, an increased latency to fall asleep, and more sleepiness during the day. Additionally they identified an increased fragmentation and a shift in diurnality, in PKU mice (197). Previously, results of the study by DeGiorgis et al. had shown a consistent delay in the maturation of trace-alternant and spindle scores in children with PKU (198). Surendran et al. identified that qualifying and addressing sleep disturbance may mitigate cognitive and psychiatric sequelae of PKU in the mouse model (199, 200).

## Conclusions

Elevated rates of psychiatric illness and cognitive impairment occur in PKU, and their nature and prevalence vary in accordance with the severity of the deficit in PAH function, the degree of dietary treatment, and—perhaps most crucially—the timing of attempts to attenuate the hyperPhe that defines the illness. The differing epochs of treatment availability and recommendations—from untreated patients, through varying duration of “early-treated” patients, through to more recent moves to ensure that “continuously treated” patients are treated lifelong—have resulted in vast differences in cognitive and psychiatric outcomes. Increasingly it is recognized that lifelong treatment offers not just the best dietary “coverage” of crucial developmental periods, but has significant implications for functional outcome and quality of life.

Although some of the effects of hyperPhe on monoaminergic function and myelination appear reversible, persistent and prolonged hyperPhe across key neurodevelopmental periods appears to cause permanent alterations to the trajectory of brain development. When this occurs across early periods of neurodevelopment, gross intellectual and behavioral changes occur.

While the emerging data show consistent elevations in rates of these disorders, very little data exist on treatment of psychiatric illness, in particular in the setting of PKU. For some off-diet patients, a return to diet may afford significant improvement in common psychiatric symptoms. It is not known, however, how likely diet alone is to effect a full remission in psychiatric illness; nor is evidence available to guide clinicians on the use of commonly utilized treatments for these disorders—such as antidepressants in mood and anxiety disorders, and stimulants in attentional disorders. Further evaluation of specific agents in these patient populations is warranted.

The broad range of neuropsychological difficulties seen in children, adolescents and adults with PKU reflects the population’s heterogeneity in overall functioning. Early treated individuals with PKU that maintain a strict low Phe diet throughout life are more likely to fulfil their academic potential, without compromise to intellectual functioning. The accumulating body of literature suggests that “diet for life” also negates the likelihood of attention, processing speed, and executive deficits in early treated adults with PKU. As a significant number of PKU individuals with cognitive inefficiencies have co-morbid mood and anxiety symptoms, future research should consider looking at the contribution of depression and anxiety on the neuropsychological profile in PKU. It is possible that enhanced psychological and pharmacological treatment of psychiatric symptoms may alleviate subtle cognitive deficits, particularly in the areas of complex attention and speed of information processing. Further research is required to look at the relationship between social cognition, psychological adjustment, and quality of life with optimal illness control.

The evidence available suggests that dietary adherence attenuates, but does not completely eliminate, the elevated risk for psychiatric illness and cognitive impairment in PKU. This may be in part because reductions in Phe down to the levels seen in non-PKU individuals are very difficult to achieve by diet alone; furthermore, the degree of protein restriction required may have other downstream effects on brain structure and function that are not yet clearly understood. It is, however, important to also acknowledge that the need to adhere to a restrictive diet, have separate “PKU foods” and supplementation, is a stressor in itself. For both pediatric and adult patients, this need for diet can be stigmatizing and socially limiting and affect a patient’s self-concept significantly. This, along with diet adherence itself, is onerous and often difficult to maintain. Treatment modalities, such as pegvaliase, that do not involve significant dietary restriction may allow PKU patients to have the best of both worlds: a relatively normal diet, free of restriction and stigma, alongside stable mental health and optimal cognitive functioning.

## Author Contributions

MW conceived and provided the methodological framework for the review and completed the manuscript. KA, SF, and WK contributed to manuscript design and writing. JP, TF, and GJ contributed to the manuscript.

## Funding

BioMarin Pharmaceutical Inc provided support to cover the cost of open-access publication, but were not involved in the development or review of this manuscript at any stage.

## Conflict of Interest Statement

The authors declare that the research was conducted in the absence of any commercial or financial relationships that could be construed as a potential conflict of interest.
